# Supplementary material for: Molecular QTL are enriched for structural variants in a cattle long-read cohort
Source: Commun Biol. 2026 Jan 21;9:290. doi: 10.1038/s42003-026-09596-w (PMC12923665; doi:10.1038/s42003-026-09596-w)
Supplement: Supplementary file 2 — Description of Additional Supplementary Files [file 42003_2026_9596_MOESM2_ESM.docx]

**Description of Additional Supplementary File**

File name: Supplementary data 1
Description: Metadata for the cohort.

File name: Supplementary data 2
Description: The source data behind the graphs in the paper.
