# Supplementary material for: Molecular QTL are enriched for structural variants in a cattle long-read cohort
Source: Commun Biol. 2026 Jan 21;9:290. doi: 10.1038/s42003-026-09596-w (PMC12923665; doi:10.1038/s42003-026-09596-w)
Supplement: Supplementary file 5 — Reporting summary [file 42003_2026_9596_MOESM5_ESM.pdf]

nature portfolio

Corresponding author(s): Hubert Pausch

Last updated by author(s): Nov 25, 2025

Reporting Summary

Nature Portfolio wishes to improve the reproducibility of the work that we publish. This form provides structure for consistency and transparency in reporting. For further information on Nature Portfolio policies, see our Editorial Policies and the Editorial Policy Checklist.

Statistics

For all statistical analyses, confirm that the following items are present in the figure legend, table legend, main text, or Methods section.

n/a	Confirmed	
☐	☒	The exact sample size (*n*) for each experimental group/condition, given as a discrete number and unit of measurement
☐	☒	A statement on whether measurements were taken from distinct samples or whether the same sample was measured repeatedly
☐	☒	The statistical test(s) used AND whether they are one- or two-sided<br>*Only common tests should be described solely by name; describe more complex techniques in the Methods section.*
☐	☒	A description of all covariates tested
☐	☒	A description of any assumptions or corrections, such as tests of normality and adjustment for multiple comparisons
☐	☒	A full description of the statistical parameters including central tendency (e.g. means) or other basic estimates (e.g. regression coefficient) AND variation (e.g. standard deviation) or associated estimates of uncertainty (e.g. confidence intervals)
☐	☒	For null hypothesis testing, the test statistic (e.g. *F*, *t*, *r*) with confidence intervals, effect sizes, degrees of freedom and *P* value noted<br>*Give P values as exact values whenever suitable.*
☒	☐	For Bayesian analysis, information on the choice of priors and Markov chain Monte Carlo settings
☒	☐	For hierarchical and complex designs, identification of the appropriate level for tests and full reporting of outcomes
☐	☒	Estimates of effect sizes (e.g. Cohen's *d*, Pearson's *r*), indicating how they were calculated

Our web collection on statistics for biologists contains articles on many of the points above.

Software and code

Policy information about availability of computer code

Data collection	We used PacBio and Illumina sequencing to generate genome data for cattle samples. This is described in full detail in material and methods. DNA and RNA sequencing data of the analysed cohort are available in the ENA database at the study accessions PRJEB42335 (Long-read sequencing data from cattle for the purpose of de-novo genome assembly), PRJEB28191 (Short read sequencing of cattle) and PRJEB46995 (Testis transcriptome of mature bulls). Accession identifiers for all samples are available as Supplementary Table 4. Gene expression and splicing matrices, a VCF file of genome-wide small and structural variant genotypes used for e/sQTL mapping, a cross-table to link genotype and transcriptome data as well as results from e/sQTL mapping have been archived at zenodo (https://zenodo.org/records/15431127).
Data analysis	The following publicly available software tools were used; all software is cited in the material and methods section.<br>minimap2 (v2.24)<br>SAMtools (v1.19.2)<br>bwa-mem2 (v2.2.1)<br>BEDtools (v2.30)<br>DeepVariant (v1.6.0)<br>GLnexus (v1.4.1)<br>pbsv (v2.9.0)<br>RepeatMasker (v.4.1.5)<br>hap.py (v0.3.15)<br>sniffles2 (v2.2)<br>TRF (v4.09.1)<br>VEP (release 113)<br>plink (v1.90b6.26)

plink (v2.00a3.6LM)
Jasmine (v1.1.5)
mosdepth (version 0.2.2)
fastp (v0.23.1)
Kallisto (v0.50.0)
tximport (v1.34.0)
STAR (version 2.7.11a)
Regtools (v0.5.2)
LeafCutter (v0.2.9)
QTLtools (v1.3.1)
Codes used in this study are available in GitHub: https://github.com/AnimalGenomicsETH/HiFi_cohort. A Code availability statement is included in the manuscript.

For manuscripts utilizing custom algorithms or software that are central to the research but not yet described in published literature, software must be made available to editors and reviewers. We strongly encourage code deposition in a community repository (e.g. GitHub). See the Nature Portfolio guidelines for submitting code & software for further information.

Data

Policy information about availability of data

All manuscripts must include a data availability statement. This statement should provide the following information, where applicable:

- Accession codes, unique identifiers, or web links for publicly available datasets
- A description of any restrictions on data availability
- For clinical datasets or third party data, please ensure that the statement adheres to our policy

DNA and RNA sequencing data of the analysed cohort are available in the ENA database at the study accessions PRJEB42335 (Long-read sequencing data from cattle for the purpose of de-novo genome assembly), PRJEB28191 (Short read sequencing of cattle) and PRJEB46995 (Testis transcriptome of mature bulls). Accession identifiers for all samples are available as Supplementary Table 4. Gene expression and splicing matrices, a VCF file of genome-wide small and structural variant genotypes used for e/sQTL mapping, a cross-table to link genotype and transcriptome data as well as results from e/sQTL mapping have been archived at zenodo (https://zenodo.org/records/15431127).

Research involving human participants, their data, or biological material

Policy information about studies with human participants or human data. See also policy information about sex, gender (identity/presentation), and sexual orientation and race, ethnicity and racism.

Reporting on sex and gender	NA
Reporting on race, ethnicity, or other socially relevant groupings	NA
Population characteristics	NA
Recruitment	NA
Ethics oversight	NA

Note that full information on the approval of the study protocol must also be provided in the manuscript.

Field-specific reporting

Please select the one below that is the best fit for your research. If you are not sure, read the appropriate sections before making your selection.

☒ Life sciences ☐ Behavioural & social sciences ☐ Ecological, evolutionary & environmental sciences

For a reference copy of the document with all sections, see nature.com/documents/nr-reporting-summary-flat.pdf

Life sciences study design

All studies must disclose on these points even when the disclosure is negative.

Sample size	We sequenced an existint biobanked cohort (N=120) with HiFi reads.<br>No specific calculations were made to pre-determine sample size.
Data exclusions	we applied standard quality control to exclude sequencing reads with low quality
Replication	NA
Randomization	NA

Blinding | NA

Reporting for specific materials, systems and methods

We require information from authors about some types of materials, experimental systems and methods used in many studies. Here, indicate whether each material, system or method listed is relevant to your study. If you are not sure if a list item applies to your research, read the appropriate section before selecting a response.

Materials & experimental systems

n/a	Involved in the study
☒	☐ Antibodies
☒	☐ Eukaryotic cell lines
☒	☐ Palaeontology and archaeology
☐	☒ Animals and other organisms
☒	☐ Clinical data
☒	☐ Dual use research of concern
☒	☐ Plants

Methods

n/a	Involved in the study
☒	☐ ChIP-seq
☒	☐ Flow cytometry
☒	☐ MRI-based neuroimaging

Animals and other research organisms

Policy information about studies involving animals; ARRIVE guidelines recommended for reporting animal research, and Sex and Gender in Research

Laboratory animals | *For laboratory animals, report species, strain and age OR state that the study did not involve laboratory animals.*

Wild animals | Tissue was obtained from biobanked samples that were collected previously by Mapel et al. (2024)

Reporting on sex | NA

Field-collected samples | NA

Ethics oversight | No animals were sampled for this study. No ethics approval was required for this study. The following ethics statement is included in the manuscript: The Bos taurus taurus tissues used in this study were previously collected from a commercial abattoir in Zürich, Switzerland after regular slaughter. None of the authors were involved in the decision to slaughter the bulls. No ethics approval was required for this study.

Note that full information on the approval of the study protocol must also be provided in the manuscript.

Plants

Seed stocks | NA

Novel plant genotypes | NA

Authentication | NA
